# Multimodality Treatment of Brain Arteriovenous Malformations with One-Staged Hybrid Operation: Clinical Characteristics and Long-Term Prognosis

**DOI:** 10.1155/2022/2559004

**Published:** 2022-02-27

**Authors:** Yuanfeng Jiang, Chaofan Zeng, Yiqun Zhang, Xiaobo Xu, Hancheng Qiu, Weijian Jiang

**Affiliations:** ^1^Medical College of Soochow University, Suzhou, China; ^2^Department of Neurosurgery, Beijing Tiantan Hospital, Capital Medical University, Beijing, China; ^3^Department of Critical Care Medicine, New Era Stroke Care and Research Institute, The PLA Rocket Force Characteristic Medical Center, Beijing, China; ^4^Department of Neurology, New Era Stroke Care and Research Institute, The PLA Rocket Force Characteristic Medical Center, Beijing, China; ^5^Department of Vascular Neurosurgery, New Era Stroke Care and Research Institute, The PLA Rocket Force Characteristic Medical Center, Beijing, China

## Abstract

**Objective:**

We aimed to evaluate the clinical characteristics and long-term prognosis of brain arteriovenous malformations (bAVMs) treated with multimodality management of one-staged hybrid operation.

**Methods:**

We identified bAVM patients treated with one-staged hybrid operation from a multicenter prospective cohort study (NCT03774017) between January 2016 and June 2020. Patients were divided into unruptured and ruptured groups by the hemorrhagic presentation. Long-term (>12 months) neurological disability, postoperative complications of stroke, and nidus obliteration were evaluated and compared between groups. Prognostic predictors associated with outcomes were analyzed.

**Results:**

A total of 130 patients were identified in the study receiving one-staged hybrid operations, including 61 unruptured cases and 69 ruptured cases. Mean age was 29.1 years old, with 78 (60.0%) being male. Patients included in the study were followed up for a mean period of 37.4 (11.07) months. The annual hemorrhagic risk was 4.2% per year. Thirteen postoperative stroke events were detected in 11 patients (8.5%). Long-term disability occurred in 6.9% of cases, and 86.2% of patients experienced an unchanged or improved neurological status at the last follow-up. All patients achieved complete obliteration on follow-up angiographies. Increased AVM volume was associated with a higher risk of postoperative stroke (odds ratio (OR) 1.021, 95% confidence interval (CI) 1.006-1.037, and *P* = 0.006). Poor neurological status (OR 6.461, 95% CI 1.309-31.889, and *P* = 0.022) and infratentorial location (OR 5.618, 95% CI 1.158-27.246, and *P* = 0.032) were independent predictors for long-term disability.

**Conclusions:**

One-staged hybrid operation of embolization combined microsurgical resection can be performed as a safe and effective strategy for bAVM treatments. Long-term prognosis of complete obliteration with low rates of morbidity and mortality can be achieved. Unruptured and ruptured bAVMs acquired similar favorable outcomes after the multimodality treatment.

## 1. Introduction

Brain arteriovenous malformations (bAVMs) are congenital anomalies of dilated arteries and veins without capillary networks, allowing high-flow arterial blood to shunt directly into the venous system [1, 2]. Intracranial hemorrhage is the most common primary manifestation with an annual bleeding rate of 2%-4%, which leads to a high risk of neurological morbidity and mortality [3]. Thus, appropriate managements of completely obliterating the lesions are necessary.

Modern treatments are available for bAVMs, including endovascular embolization, microsurgical resection, stereotactic radiosurgery alone, or in combination [4]. The integrated strategy of endovascular embolization and microsurgical resection is commonly utilized in the treatment of bAVMs as novel multimodality management in many institutions [5–8]. Endovascular embolization facilitates the subsequent microsurgery by reducing blood flows and volume for surgical safety, formatting clear resecting planes for less injury, and potentially protecting eloquent areas [9–13]. Intraoperative digital subtraction angiography (DSA) is applied to detect any residual lesions during operation [14]. Besides, the interval risks and gradient hemodynamic changes were diminished as compared with multistaged treatments. However, few studies have assessed the applicable population, preoperative embolization strategy, and the long-term outcomes of the one-staged hybrid operation for bAVM treatment. We presumed that the multimodality management can be performed as a safe and effective strategy for bAVM treatments, with low rate of morbidity and residual lesions in the long term. Therefore, we aimed to describe the clinical experience and evaluate the long-term safety and benefits of bAVMs treated with hybrid operations in this study.

## 2. Materials and Methods

### 2.1. Study Design and Participants

BAVM patients were reviewed from the database of a multicenter prospective cohort study (NCT03774017) between January 2016 and June 2020. Patients who underwent endovascular embolization combined microsurgical resection in one-staged hybrid operation were included in the study. Patients with inadequate clinical data or receiving monotherapies of embolization or microsurgery were excluded. The clinical outcomes between unruptured and ruptured bAVMs were compared. The study was approved by the Institutional Review Board (IRB) of Beijing Tiantan Hospital (KY2016-034-02). Written informed consents were obtained from all participants.

### 2.2. Data Collection

Data of demographics, personal and operation history, clinical features, and radiographic presentations were obtained. The operation history included endovascular embolization and radiosurgery. Different primary symptoms were summarized into four categories: hemorrhage, seizure, neurological dysfunction, and headache. Radiographic presentations included morphology of bAVMs, eloquent location, and angioarchitecture of lesions. The bAVMs were classified by the Spetzler-Martin (SM) grading scale, and the bAVM volume was calculated by (width × height × length)/2 [15]. The clinical outcomes and bVMs obliteration were acquired from the evaluation of discharge and follow-up. Two experienced neurosurgeons (Y.J. and C.Z.) independently evaluated the radiographic findings.

### 2.3. Treatment

The multimodality treatment of microsurgery and endovascular embolization was decided by a multidisciplinary team with neurosurgeons and neurointerventionalists involved. Preoperative embolization was conducted as an adjunctive therapy. The subsequent microsurgical resection was performed immediately in the hybrid operating room, with the assistance of neuronavigation and indocyanine fluorescence angiography (ICG). Intraoperative DSA was applied during microsurgery to confirm the complete elimination of bAVMs. The hybrid management followed the study protocol across the multicenters [16].

### 2.4. Outcome Evaluation and Follow-Up

The clinical status was assessed by the modified Rankin Scale (mRS) on admission, at discharge and in follow-up. The primary outcome was defined as the neurological disability (mRS score > 2). Postoperative stroke, defined as intracranial hemorrhage or cerebral infarction, was considered as the secondary outcome [16]. Besides, all-cause mortality and obliteration of bAVM lesions were collected. Patients were followed up in the 3^rd^, 6^th^, 12^th^ months, and annually after the operation. The short-term outcomes were obtained at discharge, and long-term outcomes were evaluated at the last follow-up.

### 2.5. Statistical Analysis

SPSS (version 26.0, IBM, NY, USA) was used for statistical analyses in the study. The categorical variables were reported as frequencies, and continuous variables were presented as mean (standard deviation (SD)) and median (interquartile range (IQR)). The Pearson Chi-square test or Fisher's exact test was used to compare categorical variables between groups. Student's *t*-test or Mann-Whitney *U* test was performed to compare continuous variables. Univariate and multivariate logistic regression analyses were conducted to identify the predictors for postoperative stroke and long-term neurological disability. Variables that achieved *P* < 0.10 in univariate analyses were included in multivariate analyses. Age and sex were adjusted in the multivariate analyses. Statistical significance was defined as *P* value < 0.05.

## 3. Results

**Figure 1:**
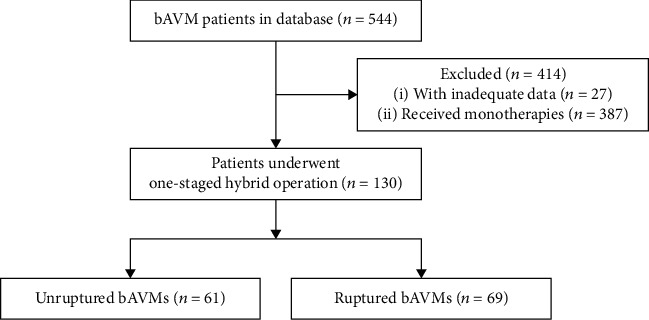
Flow diagram of the study participants. bAVMs: brain arteriovenous malformations.

Five hundred and forty-four patients were involved in the prospective multicentered cohort study. After excluding 414 patients with incomplete data or cured by monotherapies, a total of 130 bAVM patients (unruptured : ruptured = 61 : 69) receiving one-staged hybrid operation were enrolled in the study (Figure 1).

### 3.1. Baseline Characteristics

Demographic, clinical, and radiographic characteristics of unruptured and ruptured bAVM patients are summarized in Table 1. The mean age on admission was 29.1 years (range, 5-64). Sixteen cases (12.3%) had received prior interventions, including endovascular embolization in 12 (9.2%) and radiosurgery in 4 (3.1%). Hemorrhage (53.1%) manifested as the most common onset symptom, followed by seizure (32.3%), neurological dysfunction (22.3%), and headache (7.7%). Poor neurological status (mRS > 2) accounts for 7.7% of cases (*n* = 10) on admission. Thirty cases (23.1%) were high grade (IV-V). The mean size and volume of bAVM lesions were 3.9 (1.73) cm and 15.1 (26.27) cm^3^. Infratentorial locations were involved in 11 cases (8.5%), and eloquent areas were detected in 57.7% of cases (*n* = 75). Supplies of the anterior cerebral circulation and perforating arteries were identified in 86 (66.2%) and 19 cases (14.6%), respectively. 27.7% of cases (*n* = 36) had deep venous drainage. The follow-up duration was 37.4 months on average (range, 8-53). Compared with the unruptured group, patients with ruptured bAVMs tended to present poor neurological status and were less likely to exhibit seizure (*P* < 0.05 for both).

### 3.2. Clinical Outcomes

**Figure 2:**
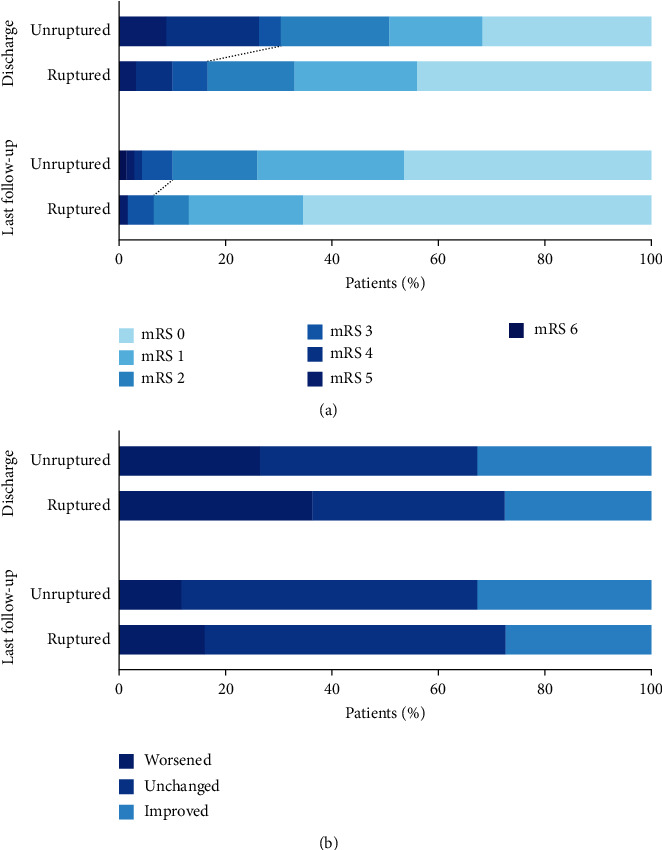
Comparison of neurological outcomes between unruptured and ruptured groups. (a) The proportions of patients with mRS scores ranging from 0 to 6 are shown for patients at discharge and last follow-up. (b) There was no significant difference in the variation of mRS score at discharge and last follow-up between groups, and worsened status occurred in 31.5% of patients at discharge and 13.8% of patients at last follow-up. mRS: modified Rankin Scale.

**Figure 3:**
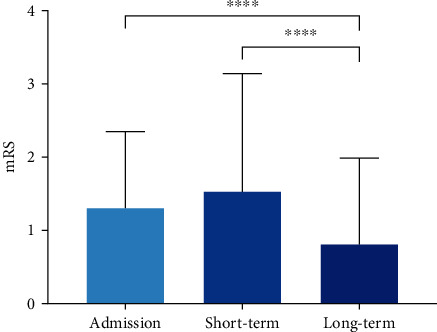
Comparison of the neurological status in different timepoints of bAVM patients. The mRS score of patients in the long-term follow-up was significantly lower than that on admission and in the short-term follow-up (^∗∗∗∗^*P* < 0.0001; ^∗∗∗^*P* < 0.001, respectively). mRS: modified Rankin Scale.

Treatment features and clinical outcomes between unruptured and ruptured bAVM patients are presented in Table 2. The annualized hemorrhagic risk for the bAVMs was 4.2% per year. On average, the duration of microsurgical resection in the hybrid operation was 5.9 hours (unruptured vs.ruptured = 5.7 (3.56) h vs.6.1 (4.03) h, *P* = 0.646). Seven (5.4%) bAVM remnants were detected by intraoperative DSA and subsequently resected. All residual lesions were confirmed as complete elimination during the third angiographies. In the postoperative period, thirteen stroke events occurred in 11 patients (8.5%), including eight intracranial hemorrhages and five cerebral infractions. Two cases experienced both types of stroke, which contributed to unfavorable outcomes. The length of stay averaged 21.1 days, with no significant intergroup difference (unruptured vs.ruptured = 20.9 (10.19) days vs.21.3 (10.80) days, *P* = 0.824). The short-term mRS score was 1.5 (1.58) on average. Though the ruptured group possessed a significantly higher mRS score at discharge (*P* = 0.019), the long-term outcomes were similar between groups (*P* > 0.05 for all). Eleven patients were disabled (6.9%) or dead (1.5%), while 119 patients (91.5%) achieved neurological deficit-free (mRS ≤ 2) at last follow-up (Figure 2(a)). In regard to the variation of mRS scores (Figure 2(b)), most patients experienced an unchanged or improved neurological status by the time of discharge (68.5%, *n* = 89) and last follow-up (86.2%, *n* = 112). The long-term mRS score was significantly lower than that on admission and in the short term. (*P* < 0.0001; *P* < 0.001, respectively) (Figure 3). In terms of the obliteration of bAVMs, there were no residual lesions detected in the follow-up angiographies.

### 3.3. Predictors for Postoperative Stroke and Long-Term Neurological Disability

In the analyses of the predictors related to clinical outcomes, the univariate analysis demonstrated that age at diagnosis, AVM maximum diameter, AVM volume, and duration of microsurgery were associated with the occurrence of postoperative stroke. After adjusting for male sex in the multivariate analysis, AVM volume (OR 1.021, 95% CI 1.006-1.037, and *P* = 0.006) remained an independent risk factor for postoperative stroke (Table 3).

Predictors for long-term neurological disability were analyzed. Univariate analysis showed that onset neurological dysfunction, poor neurological status, and infratentorial location were associated with long-term neurological disability. Poor neurological status (OR 6.461, 95% CI 1.309-31.889, and *P* = 0.022) and infratentorial location (OR 5.618, 95% CI 1.158-27.246, and *P* = 0.032) were confirmed as significant risk factors for long-term neurological disability in the age and sex adjusted multivariate logistic regression analysis (Table 4).

## 4. Discussion

One-staged hybrid operation incorporates the advantages of endovascular embolization and microsurgical resection, but the long-term prognosis has not been described. In this study, we identified the long-term safety and efficacy of bAVM patients who underwent the hybrid operation. The multimodality management can be performed as a safe and effective strategy for treating bAVMs, by achieving a low morbidity rate of 6.9% and a mortality rate of 1.5%, with complete obliteration in the follow-up. Postoperative stroke was observed in 8.5% of patients during hospitalization. Furthermore, we found that increased AVM volume was associated with a higher risk of postoperative stroke; poor neurological status and infratentorial location were correlated with a higher risk of long-term disability. The safety and efficacy of the management for the complex bAVMs has been confirmed by previous studies [5–8]. Recently, our team has also demonstrated that the suite was an efficient treatment for SM grade III-V bAVMs [17]. The preoperative embolization was capable of minimizing the surgical difficulties, and the hybrid operation had obvious shorter resection compared with the monotherapy of microsurgery. The advantages are vital for neurosurgeons with insufficient surgical experience in the operations of high-grade bAVMs or under emergency circumstances.

In the current study, the majority of patients reached long-term neurological deficit-free. The low rate of morbidity was proposed to be associated with the therapeutic preoperative embolization [10, 18]. The modalities of endovascular embolization and microsurgical resection have been used as single or combined approaches for curing bAVMs. Typically, microsurgical resection is favored as the approach to achieve a superior rate of complete obliteration with an acceptable incidence of morbidity and complications [4]. Endovascular embolization reaches a low rate of unfavorable outcomes as curative or adjuvant therapies [19]. The strategy facilitates the surgical resection by occluding the feeding arteries and degrading the bAVMs or embolizing the deep perforators that are inaccessible for microsurgeries [20–22]. Grüter et al. confirmed the safety of the combined treatment in a retrospective study of 18 bAVM patients, with a complication rate of 11% [5]. Kocer et al. performed single-stage combined treatments on 31 bAVMs with SM grades III-V [23], in which the long-term disability and mortality rate was 6.4%. It is suggested that high-grade bAVMs can be eliminated by reducing the rate of morbidity and mortality by the hybrid operation.

Although the accumulation of treatment-related risks of the combined operation has been concerned, the complications of one-staged multimodality management were comparable with single approaches. In the present study, the postoperative complications occurred in 8.5% of patients, which conformed to the previous studies with a rate of 7.2%-12.5% [8, 24]. A recent meta-analysis of treatment for bAVMs demonstrated that complications were observed in 7.4% after microsurgery and in 6.6% after embolization [20]. Brown et al. conducted the hybrid treatment in 19 bAVM cases, in which neurological outcomes were similar with staged managements without hemorrhagic events after microsurgery or embolization [6]. In our study, the clinical outcomes were similar between one-staged hybrid operation and multistaged operations (*P* = 0.269 for both). However, the stage of preoperative embolization varied across different institutions. The multistaged strategy was adopted to minimize the risk of normal perfusion pressure breakthrough (NPPB) by progressively reducing the blood flow and normalize the hemodynamics of large or high-flow bAVMs [25–27]. Nevertheless, the stepwise modality carried potential risks of hemorrhage in-between the interventions, which ranged from 5.9% to 20% [28–30]. The unfavorable outcomes may correlate with the recanalization of the nidus and the recruitment of new collateral circulation from adjacent feeding arteries after embolization [19]. Conversely, the management of one-staged hybrid operation diminished the additional treatment risks by prompt resection followed preoperative embolization [5].

The primary goal of bAVM treatment is to completely obliterate the lesions, thereby eliminating the bleeding risk and reducing morbidity or mortality. In the study by Blauwblomme et al., the recurrence occurred in 4.35% of patients following the combined embolization and surgery [31]. Other series reported the occlusion rates that vary from 95.5% to 100% after hybrid treatment [23, 32]. Similarly, the one-staged hybrid management resulted in the total eradication of lesions in our study. By routine intraoperative DSA checking, remnant lesions can be detected and resected subsequently before closure. Importantly, the complete obliteration of high-grade bAVMs highlighted the advantage of hybrid operation. Nevertheless, there may remain false-negative examination findings owing to the arterial spasm or temporary thrombosis. Thus, the delayed angiographies at 1-3 years after treatment are necessary [33].

Radiographic characteristics of bAVMs result in subtypes with different operative risks. In the present study, the AVM volume was significantly associated with postoperative complications of stroke. Previous report has revealed that the AVM size was a predictor for complications following microsurgery [24]. The larger size indicated more difficulties during surgery and higher treatment risks after the procedure. Reduction in blood flow and volume of bAVMs facilitates surgical resection. Therefore, preoperative occlusion of the volume is significantly required. The poor neurological status and infratentorial location were identified as risk factors for long-term disability. van Swieten et al. proposed that the preoperative status determined the neurological outcomes [34]. Infratentorial bAVMs were proved to carry a higher risk of worse outcomes [12, 35]. In addition, the correlation between prior hemorrhage and neurological outcomes remained controversial. Ellis et al. reported that the hemorrhagic presentation was associated with morbidity and mortality [35]. However, other studies suggested that nonhemorrhage was correlated with neurological complications [24, 36]. In our series, although more poor neurological conditions were identified in ruptured bAVMs on admission, these patients achieved equivalent long-term outcomes after hybrid operations.

There are several limitations in our study. First, it is a nonrandomized prospective study with a relatively small sample size. Second, variables including concomitant aneurysms and diffusiveness of AVM nidus were not enrolled in the study, which might result in potential bias of baseline characteristics. Third, parts of patients were followed up with magnetic resonance angiography (MRA), which might lead to false-negative detection of residual lesions. Fourth, a longer-term angiographic follow-up was needed to detect the recurrent and residual bAVMs.

## 5. Conclusions

One-staged hybrid operation of combined embolization and microsurgery can be performed as a safe and effective strategy for treating bAVMs. Long-term prognosis of complete obliteration with low rates of morbidity and mortality can be achieved by multimodality management. Unruptured and ruptured bAVMs acquired similar favorable outcomes after treatments. Increased AVM volume was associated with a higher risk of postoperative stroke. Poor neurological status and infratentorial location were independent predictors for long-term disability.

## Figures and Tables

**Table 1:** Baseline characteristics of unruptured and ruptured bAVM patients.

Variables	Total (*n* = 130)	Unruptured (*n* = 61)	Ruptured (*n* = 69)	*P* value
Age (mean (SD)), y	29.1 (13.24)	29.9 (12.29)	28.3 (14.08)	0.507^†^
Sex, male (%)	78 (60.0)	40 (65.6)	38 (55.1)	0.223^‡^
Prior treatments (%)				
Embolization	12 (9.2)	2 (3.3)	10 (14.5)	0.057^‡^
Primary symptom (%)				
Hemorrhage	69 (53.1)	0 (0)	69 (100.0)	<0.001^∗^^§^
Seizure	42 (32.3)	38 (62.3)	4 (5.8)	<0.001^∗^^‡^
Neurological dysfunction	29 (22.3)	9 (14.8)	20 (29.0)	0.052^‡^
Headache	10 (7.7)	5 (8.2)	5 (7.2)	>0.999^‡^
Admission mRS score (%)				
Mean (SD)	1.3 (1.04)	1.1 (0.66)	1.5 (1.26)	0.012^∗^^†^
Good (0-2)	120 (92.3)	61 (100.0)	59 (85.5)	0.006^∗^^§^
Poor (3–5)	10 (7.7)	0 (0)	10 (14.5)	
Spetzler-Martin grade (%)				0.537^||^
I	10 (7.7)	3 (4.9)	7 (10.1)	
II	39 (30.0)	21 (34.4)	18 (26.1)	
III	51 (39.2)	26 (42.6)	25 (36.2)	
IV	25 (19.2)	9 (14.8)	16 (23.2)	
V	5 (3.8)	2 (3.3)	3 (4.3)	
AVM morphology and angioarchitecture (%)				
Maximum diameter (median (IQR)), cm	3.9 (1.73)	4.0 (1.40)	3.7 (2.00)	0.278^||^
Volume (median (IQR)), cm^3^	15.1 (26.27)	19.5 (24.05)	10.9 (28.25)	0.124^||^
AVM location				
Supratentorial	119 (91.5)	56 (91.8)	63 (91.3)	0.919^‡^
Infratentorial	11 (8.5)	5 (8.2)	6 (8.7)	
Eloquence	75 (57.7)	30 (49.2)	45 (65.2)	0.065^‡^
Anterior circulation involvement	86 (66.2)	41 (67.2)	45 (65.2)	0.810^‡^
Deep perforator supply	19 (14.6)	8 (13.1)	11 (15.9)	0.649^‡^
Deep venous drainage	36 (27.7)	14 (23.0)	22 (31.9)	0.256^‡^
Follow-up (mean (SD)), month	37.4 (11.07)	36.6 (11.51)	38.1 (10.70)	0.458^†^

SD: standard deviation; SRS: stereotactic radiosurgery; mRS: modified Rankin Scale; AVM: arteriovenous malformation; IQR: interquartile range. ^†^Student's *t*-test. ^‡^Pearson Chi-square test. ^§^Fisher's exact test. ^||^Mann-Whitney *U* test. ^∗^*P* < 0.05, significant difference.

**Table 2:** Comparison of clinical outcomes between unruptured and ruptured bAVM patients.

Variables	Total (*n* = 130)	Unruptured (*n* = 61)	Ruptured (*n* = 69)	*P* value
Duration of microsurgery (mean (SD)), h	5.9 (3.80)	5.7 (3.56)	6.1 (4.03)	0.646^†^
Length of stay (mean (SD)), d	21.1 (10.48)	20.9 (10.19)	21.3 (10.80)	0.824^†^
Postoperative stroke (%)	11 (8.5)			
Intracranial hemorrhage	8 (6.2)	2 (3.3)	6 (8.7)	0.359^‡^
Cerebral infarction	5 (3.8)	2 (3.3)	3 (4.3)	>0.999^‡^
Short-term outcomes (%)				
mRS score (mean (SD))	1.5 (1.58)	1.2 (1.41)	1.8 (1.73)	0.019^∗^^†^
Neurological disability	31 (23.8)	10 (16.4)	21 (30.4)	0.061^‡^
Long-term outcomes (%)				
mRS score (mean (SD))	0.8 (1.17)	0.6 (1.10)	0.9 (1.21)	0.113^†^
Neurological disability	9 (6.9)	3 (4.9)	6 (8.7)	0.617^‡^
Mortality	2 (1.5)	1 (1.6)	1 (1.4)	>0.999^§^
Obliteration	130 (100.0)	61 (100.0)	69 (100.0)	>0.999^‡^

SD: standard deviation; bAVM: brain arteriovenous malformation. ^†^Student's *t*-test. ^‡^Pearson Chi-square test. ^§^Fisher's exact test. ^∗^*P* < 0.05, significant difference.

**Table 3:** Logistic regression analysis for postoperative stroke.

Variables	Univariate analysis			Multivariate analysis^†^		
	OR	95% CI	*P* value	OR	95% CI	*P* value
Age	1.047	0.999-1.097	0.055	1.049	0.994-1.107	0.081
Male sex	2.878	0.798-10.383	0.106	0.236	0.055-1.010	0.052
Onset symptom						
Hemorrhage	1.609	0.447-5.787	0.467			
Seizure	0.439	0.091-2.128	0.307			
Neurological dysfunction	2.149	0.582-7.925	0.251			
Poor neurological status	3.083	0.568-16.741	0.192			
AVM location						
Supratentorial	Ref	Ref	Ref			
Infratentorial	1.090	0.126-9.407	0.938			
AVM maximum diameter	1.723	1.106-2.685	0.016	1.452	0.899-2.346	0.884
AVM volume	1.021	1.006-1.037	0.006	1.021	1.006-1.037	0.006^∗^
Eloquence	2.070	0.523-8.189	0.300			
Deep venous drainage	2.366	0.674-8.302	0.179			
Duration of microsurgery	1.119	1.003-1.247	0.043	1.084	0.945-1.243	0.356

OR: odds ratio; CI: confidence interval; AVM: arteriovenous malformation. ^†^The multivariate model was adjusted for male sex. ^∗^*P* < 0.05, significant difference.

**Table 4:** Logistic regression analysis for long-term neurological disability.

Variables	Univariate analysis			Multivariate analysis^†^		
	OR	95% CI	*P* value	OR	95% CI	*P* value
Age	1.001	0.955-1.049	0.972	1.004	0.956-1.053	0.883
Male sex	1.277	0.369-4.422	0.700	0.890	0.234-3.379	0.864
Onset symptom						
Hemorrhage	1.609	0.447-5.787	0.467			
Seizure	0.769	0.193-3.061	0.710			
Neurological dysfunction	3.299	0.928-11.728	0.065	1.928	0.447-8.316	0.375
Poor neurological status	6.000	1.298-27.735	0.022	6.461	1.309-31.889	0.022^∗^
AVM location						
Supratentorial	Ref	Ref	Ref	Ref	Ref	Ref
Infratentorial	5.203	1.151-23.517	0.032	5.618	1.158-27.246	0.032^∗^
Maximum diameter	1.270	0.814-1.980	0.292			
AVM volume	1.012	0.998-1.027	0.103			
Eloquence	2.070	0.523-8.189	0.300			
Deep venous drainage	0.977	0.244-3.909	0.974			
Duration of microsurgery	1.018	0.878-1.181	0.814			

OR: odds ratio; CI: confidence interval; AVM: arteriovenous malformation. ^†^The multivariate model was adjusted for age and male sex. ^∗^*P* < 0.05, significant difference.

## Data Availability

The data used to support the findings of this study are available from the corresponding author upon request.
